# Evaluation of the cytotoxic and mutagenic potentials of ethanolic extract of *Baccharis gaudichaudiana *DC., (Asteraceae)

**DOI:** 10.1186/1753-6561-8-S4-P27

**Published:** 2014-10-01

**Authors:** Lorraine Polloni, Aline Richter, Fernanda Croisfelt, Flávia Moreira-Vieira, Isabela De Lima, Maria De Sá, Marcus Chaves, Sofia Antunes-Miranda, Thalita Silva, Vinicius Rodovalho, Mário Spanó, Alexandre Rezende

**Affiliations:** 1 Discente do Curso de Biotecnologia da Universidade Federal de Uberlândia, MG, Uberlândia, Brazil; 2 Laboratório de Mutagênese, Instituto de Genética e Bioquímica, Universidade Federal de Uberlândia, MG, Uberlândia, Brazil

## Background

Evidenced by their traditional use and through scientific studies, natural products have been important participants in drug discovery, providing novel structures that can be used as potential drugs. Among the plants investigated to date, those of the *Baccharis *genus are important sources of natural medicinal products. Comprising more than 500 species distributed throughout the North and South American continents, this genus is commonly used in folk medicine as antipyretic agents, antirheumatic and to control hyperglycemia. *B. gaudichaudiana*, popularly known as "carquejeira-doce" is found in grasslands, Brazilian savannas, and less pronounced in tropical humid lowland. Many secondary metabolites have been characterized and isolated, such as flavonoids, diterpenes, tannins, saponins and essential oils.

## Methods

In order to evaluate the cytotoxic and mutagenic potentials of the ethanolic extract of *B. gaudichadiana*, the Somatic Mutation and Recombination Test (SMART) in wing somatic cells of *Drosophila melanogaster*, was performed employing two genetic markers located on the left arm of chromosome 3: multiple wing hairs (*mwh*, 3-0.3) - a homozygous-viable recessive mutation that produces multiple trichomes per cell instead of one trichome; and flare3 (*flr3*, 3-38.8) - a recessive mutation that produces flare-shaped wing hairs. Three *D. melanogaster *strains were used: 1) multiple wing hairs: *y; mwh j*; 2) flare-3: *flr3/In (3LR)TM3, ri ppsep l(3)89Aa bx34ee BdS*; and 3) ORR; *flare-3: ORR; flr3/In(3LR)TM3, ri ppsep l(3)89Aa bx34ee BdS*. Two different crosses were carried out: Standard (ST) cross and High-Bioactivation (HB) cross. For the ST cross, virgin flare-3 females were mated with mwh males. For the HB cross, which is characterized by an augmented level of CYP 450, virgin ORR, flare-3 females were mated with mwh males. Third instar larvae obtained from both crosses were fed chronically (48 h) with ethanolic extract of *B. gaudichaudiana *(5, 10, 20, 40 or 80 mg/mL). Ultrapure water (MilliQ) was used as negative control and urethane (10 mM) as positive control.

## Results and conclusions

The results from both crosses were rather similar. Concentrations between 5 to 40 mg/mL of the extract did not show cytotoxic activity. On the other hand, concentrations higher than 80 mg/mL significantly reduced the survival rates. The concentrations tested are not mutagenic in ST cross. However in HB cross, at higher concentration (40 mg/mL), the frequency of mutant spots was statistically increased. These results suggest that, under these experimental conditions, the ethanolic extract (at higher concentrations) might have compounds that, when metabolized by CYP450, can be mutagenic, and at high concentrations (>80 mg/mL), cytotoxic. However, further studies are needed to identify the constituents of this extract and confirm the efficacy and/or risks of its use, as well as to encourage the rational use of natural resources.
